# Cardiorespiratory fitness improves prediction of mortality of standard cardiovascular risk scores in a Latino population

**DOI:** 10.1002/clc.23427

**Published:** 2020-07-21

**Authors:** Mónica Acevedo, Giovanna Valentino, María José Bustamante, Lorena Orellana, Marcela Adasme, Fernando Baraona, Ramón Corbalán, Carlos Navarrete

**Affiliations:** ^1^ División de Enfermedades Cardiovasculares, Facultad de Medicina Pontificia Universidad Católica de Chile Santiago Chile; ^2^ Carrera de Nutrición y Dietética, Departamento de Ciencias de la Salud, Facultad de Medicina Pontificia Universidad Católica de Chile Santiago Chile; ^3^ Departamento de Matemáticas, Facultad de Ciencias Universidad de la Serena La Serena Chile

**Keywords:** cardiorespiratory fitness, cardiovascular disease, cardiovascular risk assessment, mortality, predictors

## Abstract

**Background:**

Cardiorespiratory fitness (CRF) is a powerful predictor of mortality. This study evaluated the predictive value of CRF for mortality in Chilean subjects without atherosclerotic disease compared with the Framingham, European Systematic Coronary Risk Evaluation (SCORE), and 2013 ACC/AHA risk scores and determined the incremental predictive value of CRF when added to these scores.

**Hypothesis:**

CRF improves prediction of all‐cause and cardiovascular disease (CVD)‐related mortality of the standard international risk scores.

**Methods:**

Cross‐sectional study, which evaluated 4064 subjects between 2002 and 2016. Cardiovascular (CV) risk factors, anthropometric and biochemical parameters, and blood pressure were measured. CRF was determined by metabolic equivalents during maximum stress test. The Framingham, SCORE, and ACC/AHA risk scores were calculated for all subjects. After a median follow‐up of 9 years, all‐cause and CVD‐related mortality were assessed. Receiver operating curves were built to determine mortality prediction for CRF, the risk scores, and CRF added to the scores.

**Results:**

As of August 2016, 99 deaths were reported, 33 of which were CVD‐related. All risk scores and CRF predicted CVD‐related mortality, with CRF identified as the best predictor: CRF: C = 0.88 (95% CI: 0.82‐0.93) vs Framingham: C = 0.68 (95% CI: 0.60‐0.76), SCORE: C = 0.76 (95% CI: 0.70‐0.83), and ACC/AHA: C = 0.79 (95% CI: 0.73‐0.85). Predictive power of the three scores improved when CRF was added to the model, but this was only significant for the Framingham score.

**Conclusions:**

CRF is a good predictor of both, all‐cause and CV mortality and a better predictor of CVD‐related deaths than standard risk scores in this population.

## 1 INTRODUCTION

Cardiovascular disease (CVD) remains the leading cause of death worldwide including for Chile.^1^ Therefore, risk prediction scores for the general population are essential tools for clinical decision‐making about lifestyle and pharmacologic interventions in the primary prevention setting.

The Framingham risk score, published in 1998 by Wilson et al^2^, has been one of the most widely used risk scores for the prediction of cardiovascular (CV) events.^3^ This system considers the main CV risk factors (RFs) and only assesses the risk of the hard endpoints of cardiac death and nonfatal myocardial infarction at 10 years. The European Systematic Coronary Risk Evaluation (SCORE) is another risk assessment tool used globally which estimates 10‐year risk of CV mortality and it is based on cohort studies from 12 European countries.^4^

In 2013, the American College of Cardiology (ACC)/American Heart Association (AHA) proposed the Pooled Cohort Atherosclerotic Cardiovascular Disease (ASCVD) Risk Equation, which determines the 10‐year risk of ASCVD events.^5^ This system includes the same variables as the Framingham score but adds race to the equation and cerebrovascular disease to the hard endpoints. This score has been criticized, because it classifies a large proportion of individuals as “higher risk,” potentially overestimating when compared with observed event rates.^6^, ^7^ However, the ACC/AHA score has attempted to address criticisms of the Framingham score about the underestimation of risk in young people and women.^8^ In a previous study, our group demonstrated the superiority of the ACC/AHA score over the Framingham global risk score in the prediction of mortality in a sample of Chilean subjects who attended a primary prevention program.^9^

Although these scores provide guidance for assessing CVD risk, most ASCVD events occur in people at intermediate risk, who would not merit more intense intervention according to these scores.^2^, ^4^, ^5^, ^10^ The identification of markers that might improve prediction of mortality risk and CVD events beyond traditional RFs has been increasingly used.^11^, ^12^ Indeed, recent American and European cholesterol guidelines for statin use recommend using noninvasive CV imaging techniques, such as coronary artery calcification (CAC) for patients with intermediate risk in whom the risk/benefit of using a statin is not clear.^13^, ^14^ American guidelines also recommend considering risk enhancers, such as metabolic syndrome, inflammatory markers, and chronic diseases, when assessing CV risk.^13^

Cardiorespiratory fitness (CRF) is a potent predictor of morbidity and mortality in the general population: an increase in exercise capacity of 1 metabolic equivalent (MET) produced a 15% lower risk of CV mortality.^15^, ^16^, ^17^, ^18^ However, neither exercise capacity nor other alteration in exercise stress testing (e.g., chronotropic response) has been considered in clinical guidelines to better determine CVD risk. Framingham, SCORE, or the last ACC/AHA risk scores have not integrated any parameter of exercise stress testing in the assessment of CV risk. This is surprising, as the most used CV test in the cardiology practice is exercise stress testing. It is easy to do, readily available in every cardiology ambulatory clinic, and cheap.^19^ Moreover, it does not require complementary expertise for a cardiologist or internist, as ultrasound plaque determination and CAC do, and it does not radiate. It is widely known that a low exercise capacity has a significant inverse association with CV mortality.^20^

Therefore, the objective of this study was to determine the predictive value of CRF for CV mortality and its incremental predictive value when added to the Framingham, SCORE, and ACC/AHA risk scores in a Chilean population without known atherosclerotic disease.

## 2 METHODS

### 2.1 Subjects

This study had a cross‐sectional design and included a sample of 4064 subjects (35% women, mean age = 52 ± 13 years) who were evaluated in a preventive cardiology program of a university hospital between 2002 and 2016 in urban Santiago, Chile. This program only receives subjects in primary prevention, older than 18 years old, and without known ischemic heart or cerebral disease, heart failure (HF), peripheral and carotid ischemic vascular disease, and any cardiac surgery, congenital heart disease, and pregnant women.

#### 2.1.1 Data collection

Upon enrollment, all subjects participated in an interview with the program nurse, during which demographic information and medical history were collected. Also, the following variables were measured: weight, height, body mass index (BMI), waist/hip circumference, and systolic and diastolic blood pressure (SBP). In all patients, fasting venous blood samples were collected to measure glycemia, lipids, plasma creatinine levels, and thyroid‐stimulating hormone. Blood pressure was measured seated three times after 5 minutes resting at 2‐minute intervals, using a device with a brachial cuff with automatic inflation (Omron HEM 742). Subjects who performed leisure‐time physical activity <1 time/week of <30 minutes were considered sedentary.

Cardiovascular RFs were defined according to the following criteria: (a) hypertension: previous medical diagnosis of hypertension, with or without pharmacologic treatment, and subjects with two or more BP determinations ≥140/90 mm Hg on alternate days; (b) dyslipidemia: previous medical diagnosis of hypercholesterolemia, with or without pharmacologic treatment, and subjects with total cholesterol ≥200 mg/dL and/or high‐density lipoprotein cholesterol (HDL‐C) < 40 mg/dL in men and < 50 mg/dL in women, respectively; (c) diabetes: was defined as fasting glucose ≥126 mg/dL, or nonfasting glucose ≥200 mg/dL, or reported diagnosis of diabetes coupled with the use of glucose‐lowering medication; (d) overweight and obesity were defined as the World Health Organization (WHO) criteria: ≥25 kg/m^2^ and ≥ 30 kg/m^2^, respectively; and (e) smoking: if the subject had smoked one cigarette or more during the last month.

#### 2.1.2 Cardiorespiratory fitness

CRF was recorded as the maximal aerobic capacity expressed in METS achieved in a symptom‐limited exercise stress test, walking on a treadmill using the Bruce protocol, according to recommendations of the AHA.^21^ For the statistical analysis, CRF was divided into tertiles. *In those few patients whom the nurse and the cardiologist thought they would not be able to walk to maximum, we used the modified Bruce protocol*.

Framingham, SCORE, and ACC/AHA risk scores were calculated for all subjects according to published equations.^2^, ^4^, ^5^ At a mean follow‐up of 9 ± 4 years, all‐cause and CVD‐related mortality (CV and cerebrovascular) were determined in August 2016 by consulting the death certificates in the Chilean Civil Registry. Since there is no reliable national registry for nonfatal CV and cerebrovascular events in the country, only total and CV mortality were recorded. We considered CV deaths when the certificates included any CV disease/events (e.g., acute myocardial infarction, coronary heart disease, stroke, heart failure, arrhythmia, and ischemia) or cardiorespiratory arrest without a non‐CVD‐related cause. We considered non‐CV deaths those that included other conditions (e.g., sepsis, trauma, cancer, infection) with or without cardiorespiratory arrest as the cause of death.

All subjects provided a written informed consent approved by the Ethics Committee of the Pontificia Universidad Católica de Chile to analyze their data in academic investigations.

#### 2.1.3 Laboratory measurements

Samples for glycemia and lipid profile were obtained by venous puncture following 12 hours of fasting. Samples for glucose measurement were collected in gray cap tubes with sodium fluoride and potassium oxalate as inhibitors of Becton Dickinson brand glycolytics: BD Vacutainer tubes. Samples for the lipid profile were collected in tubes with separating gel of the brand Becton Dickinson: BD Vacutainer SST. Glycemia and lipids were analyzed using Cobas from Roche 8000 modular analyzer series (Hitachi, Tokyo‐Japan). Low‐density lipoprotein cholesterol (LDL‐C) was calculated using the Friedewald formula when triglycerides were < 400 mg/dL.

### 2.2 Statistical analysis

Student *t*‐test for different variances with Welch correction for continuous variables and Chi‐square test for discrete variables were used to compare differences between groups. Comparisons between living and deceased and between CRF groups were adjusted by age and sex (lineal regression or Cox regression).

Discrimination models were built for the prediction of mortality risk using CRF adjusted for age, sex, and the Framingham, SCORE, and ACC/AHA risk scores. The following models were recorded: CRF; Framingham + CRF; SCORE + CRF; ACC/AHA + CRF; Framingham; SCORE; and ACC/AHA. The discriminatory capacity of these models was evaluated by the construction of receiver operating characteristic curves (ROC) with areas under the corresponding curves and 95% confidence intervals (CI) using mortality (CVD‐related) vs nonmortality as a hard endpoint. The ROC curves were based on logistic regression models, and the CIs for the area under the curve (C) were estimated using Bootstrap.^22^ A value of C = 0.50 implies a predictive value equal to chance or “nondiscrimination.” The R 2.14 software was used for the complete statistical analysis.

We also calculated net reclassification index (NRI) to determine the improvement in prediction of CVD‐related mortality when adding CRF to each risk score.^23^, ^24^

## 3 RESULTS

Table 1 shows the demographic data, prevalence of traditional RFs, CRF, and the risk scores of the study population. Subjects were middle‐aged with high rates of dyslipidemia, physical inactivity, overweight, and obesity. The prevalence of smoking was 21%. The mean CRF of the sample was 12 METS and was significantly higher in men compared with women (13 vs 10 METS; *P* < .0001). Framingham risk score was significantly higher in men vs women. No significant differences were observed between men and women for total and CVD‐related mortality rates.

**TABLE 1:** Clinical characteristics, cardiovascular risk factors, and cardiovascular risk scores of the study population by sex

	Total (n = 4064)	Men (n = 2655)	Women (n = 1409)	*P*‐value
Age, y	52 ± 13	50 ± 12	55 ± 12	.05
Follow‐up, y	9 ± 4	9 ± 4	9 ± 4	NS
Hypertension, %	30	29	31	NS
Diabetes, %	5	5	4	.09
Dyslipidemia, %	76	76	76	NS
Smoking, %	21	21	22	NS
Sedentary, %	71	70	72	NS
Overweight, %	50	57	39	<.0001
Obesity, %	20	22	16	<.0001
Cardiorespiratory fitness, METS	12 ± 3	13 ± 3	10 ± 3	.02
Framingham score, %	7 ± 6	8 ± 6	4 ± 3	.03
European SCORE, %	2 ± 3	2 ± 3	3 ± 3	.07
ACC/AHA score, %	7 ± 8	7 ± 8	6 ± 8	.09
Total mortality, %	2.4	2.7	1.8	NS
CVD‐related mortality, %	0.8	0.9	0.6	NS

*Note:* Values are expressed as mean ± SD or percentages.

Abbreviations: ACC, American College of Cardiology; AHA, American Heart Association; CVD, cardiovascular disease; METS, metabolic equivalents; NS, not significant; SCORE, Systematic Coronary Risk Evaluation.

Table 2 provides clinical characteristics, risk scores, and mortality according to tertiles of CRF. Subjects with higher aerobic capacity (ie, METs ≥13.4) were significantly younger, had lower waist circumference and SBP, blood sugar, LDL‐C, non‐HDL‐C, triglycerides, and high‐sensitivity C‐reactive protein (CRP), as well as higher HDL‐C. Also, all three risk scores were significantly lower in subjects with higher CRF (*P* < .01).

**TABLE 2:** Clinical characteristics by CRF tertiles (as determined by metabolic equivalents (mean ± SD or percentages)

	METS <10.1 (mean ± SD or %)	METS ≥10.1 and < 13.4 (mean ± SD or %)	METS ≥13.4 (mean ± SD or %)	*P* value
TOTAL	1441	1740	883	
Men	611	1266	778	
Women	830	474	105	
Age, y	60 ± 11	49 ± 11	43 ± 10	<.01
Waist, cm	93 ± 13	93 ± 12	91 ± 10	<.0001^a^
SBP, mm Hg	128 ± 15	121 ± 13	117 ± 10	<.0001^a^
Glycemia, mg/dL	97 ± 25	92 ± 13	89 ± 11	<.0001^a^
LDL‐C, mg/dL	125 ± 35	127 ± 35	121 ± 35	<.001^a^
Non‐HDL‐C, mg/dL	154 ± 40	156 ± 40	147 ± 42	<.0001^a^
Triglycerides, mg/dL	145 ± 88	146 ± 107	130 ± 94	<.0001^a^
HDL‐C, mg/dL	55 ± 16	51 ± 14	52 ± 14	<.0001^a^
CRP, mg/L	2.3 ± 2	1.8 ± 2	1.5 ± 2	<.0001^a^
Framingham score, %	8 ± 7	7 ± 6	5 ± 4	.01
European SCORE, %	4 ± 4	2 ± 2	0.7 ± 1	<.01
ACC/AHA score, %	11 ± 11	6 ± 6	3 ± 3	<.01
All‐cause mortality, n (%)	75 (5.2%)	24 (1.3%)	0 (0%)	<.001^b^
CVD‐related mortality, n (%)	26 (1.8%)	7 (0.4%)	0 (0%)	.03^b^

*Note:* Values expressed as mean ± SD or percentages.

Abbreviations: ACC/AHA, American College of Cardiology/American Heart Association; CRF, cardiorespiratory fitness; CRP, C‐reactive protein; CVD, cardiovascular disease; HDL‐C, high‐density lipoprotein; LDL‐C, low‐density lipoprotein cholesterol; METS, metabolic equivalents; NS, not significant; SBP, systolic blood pressure; SCORE, Systematic Coronary Risk Evaluation, *P* > .05.

^a^ Linear regression, adjusted for sex and age.

^b^ Cox regression, adjusted by sex and age.

Subjects with lower CRF (<10.1 METs) had significantly higher all‐cause mortality (*P* < .001) and CVD‐related mortality (*P* = .03).

A total of 99 deaths were reported from all causes, 33 of which were CVD‐related, with no significant differences between sexes. Individuals whose deaths were CVD‐related were significantly older, had higher blood glucose, and lower CRF than living subjects (Table 3). Moreover, subjects who died from CVD had significantly higher risk scores (*P* < .0001 for all).

**TABLE 3:** Clinical characteristics (± SD or percentages) for subjects who died of CVD‐related causes and other subjects

	Living Subjects (mean ± SD or %)	CVD‐related Deceased Subjects (mean ± SD or %)	*P* value
N	3965	33	
Age, y	51 ± 12	70 ± 11	<.0001
Waist, cm	92 ± 12	96 ± 13	NS^a^
BMI, kg/m^2^	27 ± 4	27 ± 4	NS^a^
SBP, mm Hg	122 ± 14	137 ± 14	NS^a^
Glycemia, mg/dL	93 ± 18	104 ± 32	.02^a^
LDL‐C, mg/dL	125 ± 36	124 ± 32	NS^a^
HDL‐C, mg/dL	53 ± 14	53 ± 15	NS^a^
Non‐HDL‐C, mg/dL	153 ± 41	155 ± 35	NS^a^
CRP, mg/L	1.9 ± 2	2.6 ± 2	NS^a^
Cardiorespiratory fitness, METS	12 ± 3	8 ± 3	.03^a^
Framingham score, %	7 ± 6	14 ± 11	<.0001
European SCORE, %	2 ± 3	9 ± 6	<.0001
ACC/AHA score, %	7 ± 8	28 ± 17	<.0001

Abbreviations: ACC/AHA, American College of Cardiology/American Heart Association; BMI, body mass index; CRP, C‐reactive protein; CVD, cardiovascular disease; HDL‐C, high‐density lipoprotein; LDL‐C, low‐density lipoprotein cholesterol; METS, metabolic equivalents; NS, not significant; SBP, systolic blood pressure; SCORE, Systematic Coronary Risk Evaluation, *P* > .05.

^a^ Cox regression, adjusted for sex and age.

**FIGURE 1:**
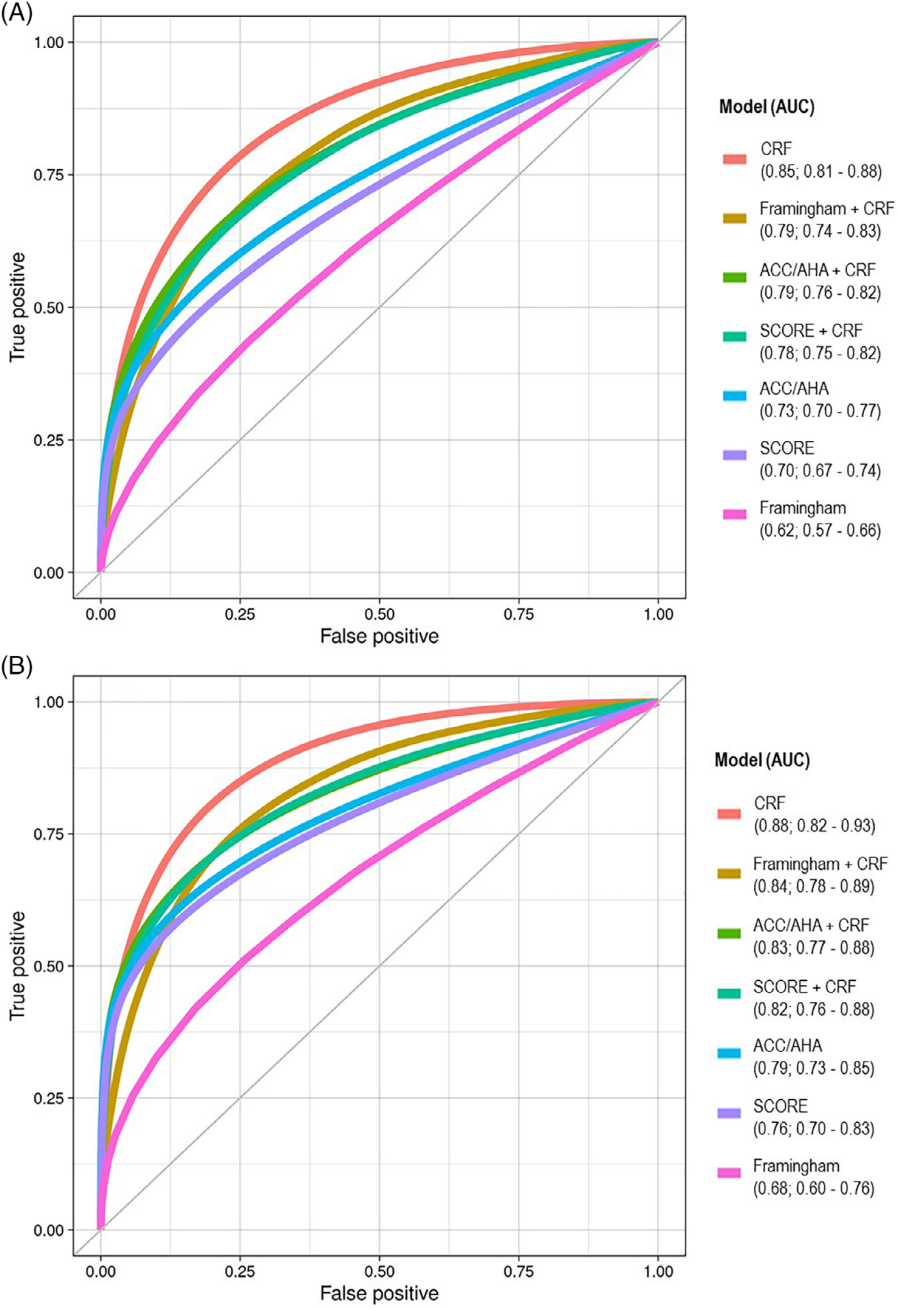
Receiver operating characteristics curves for cardiorespiratory fitness (adjusted for age and sex) and the risk prediction scores for A, total mortality and B, CVD‐related mortality. ACC/AHA, American College of Cardiology/American Heart Association; CRF, cardiorespiratory fitness; CVD, cardiovascular disease; SCORE, Systematic Coronary Risk Evaluation

CRF had the highest C‐index for both all‐cause (C = 0.85; 95% CI, 0.81‐0.88) and CVD‐related mortality (C = 0.88; 95% CI, 0.82‐0.93) and it improved the predictive value of the three risk scores but only was statistically significant for the Framingham risk score (Figure 1).

Improvement in prediction of CVD‐related mortality when adding CRF to each risk score was determined by NRI: (intervals are significant if both limits are > 0).

a) Framingham + CRF vs Framingham:

- NRI_e_ > 0 = 0.80 (0.45, 1.00); NRI_ne_ > 0 = 0.03 (−0.03, 0.11)

b) SCORE + CRF vs SCORE:

- NRI_e_ > 0 = 0.64 (0.20, 1.00); NRI_ne_ > 0 = 0.03 (−0.03, 0.08)

c) AHA/ACC + CRF vs AHA/ACC:

- NRI_e_ > 0 = 0.50 (−0.25, 1.00); NRI_ne_ > 0 = 0.05 (−0.03, 0.01)

Where: NRI_e_ is the net proportion of events assigned a higher risk or risk category (CVD‐related deaths) and NRI_ne_ is the net proportion of non‐events assigned a lower risk or risk category (non‐dead).

## 4 DISCUSSION

In this study of a middle‐aged population, CRF proved to be the best predictor of all‐cause and CVD‐related mortality followed by the ACC/AHA, SCORE, and Framingham risk scores. These results highlight the value of CRF as a significant predictor of prognosis in CVD death in middle‐aged and intermediate‐risk populations. Moreover, NRI confirmed a significant improvement in the risk classification of SCORE and Framingham when adding CRF to the models in this population.

Several studies have shown an inverse, graded, and consistent relationship between CRF levels and mortality risk.^15^, ^17^, ^19^, ^20^, ^25^ Moreover, some studies have reported that a low physical capacity is associated with a high risk of total and CVD‐related mortality.^20^, ^26^ By contrast, we have previously shown that a high CRF (≥ 10 METS), regardless of age and sex, is associated with a better CV risk factor profile and risk score.^27^ Here, we have demonstrated that CRF by itself had a good predictive ability for CVD‐related mortality, which was even better than the last ACC/AHA risk prediction. In the same way, in 2016, Wickramasinghe^20^ demonstrated in a large cohort with no prior CVD, that the presence of low fitness was associated with an increased 30 year risk for CVD‐related deaths across all ages, sex, and risk factor strata. Recently, Mansager et al^28^ reported peak estimated METs was significantly and inversely associated with all‐cause mortality. The mortality risk of reduced performance on exercise stress testing was even better than some traditional risk factors, such as diabetes and smoking. Importantly, there was no upper limit of benefit of increased aerobic fitness.

Recently, Ekblom et al^29^ confirmed an inverse association of CRF with advanced coronary atherosclerosis defined as a CAC score of 100 or higher. This information is crucial as the last American and European guidelines suggested the use of CAC to decide if a subject in intermediate‐risk deserved statin medication. In this regard, Ekblom et al hypothesized that CRF carries a real protective effect on atherosclerosis beyond conventional risk factors. A recent study of Radford et al^30^, ^31^ found that when CAC and CRF were considered together, there was a stronger inverse association between CRF and annual total CVD incidence rates as CAC burden increased. CRF and CAC would have in common that both are significantly associated with CV risk factors.^31^ Based on this evidence, one could argue that the use of CRF or CAC could give similar CV risk prediction.

Aerobic exercise has several cardioprotective effects besides reducing total and CVD‐related mortality: it increases exercise tolerance, decreases cardiac symptoms, has positive effects on lipids, and improves psychosocial factors, such as anxiety, anger, and stress, among others.^32^, ^33^ Moreover, it has an impact on lipids (increases HDL‐C and reduces triglycerides), improves insulin sensitivity, and modestly modifies body weight and fat mass, reducing the risk of developing type 2 diabetes.^32^, ^33^ Also, it positively influences BP and inflammatory and hemostatic factors (reduces CRP, increases plasma fibrinolytic activity, and reduces fibrinogen levels).^34^ We have previously demonstrated that there is less inflammation assessed by CRP levels in those with higher CRF even in the presence of the metabolic syndrome^35^ ^.^

Despite the previous beneficial effects of CRF on CVD‐related mortality and risk factors, neither American nor European cardiology associations have included CRF to current guidelines to determine CV risk better. The exclusion has been partially explained because an essential mechanism through which exercise influences its cardioprotective effects is the modification of traditional and novel risk factors. Nevertheless, Mora et al^36^ showed that the association between higher levels of physical activity and lower CVD rates could explain up to 59% of the activity‐related reduction in CVD. Inflammatory/hemostatic biomarkers made the most substantial contribution to lowered risk, followed by blood pressure, lipids, and BMI. The remaining ~40%, however, has persisted without clear explanations. The genetic background could participate, but certainly, some other exercise‐related factors still unknown could be implicated as well, and deserve more investigation.

Although Framingham, SCORE, and ACC/AHA risk scores are widely used in observational studies to predict CVD risk, the highest proportion of CVD events, including CVD‐related mortality, occurs in intermediate‐risk populations, not in individuals defined as high risk by these scores.^13^, ^14^, ^37^ Therefore, there has been increasing research for new risk markers to refine or complement the discriminative capacity of current risk scoring systems. To date, the determination of CAC emerged as the most important predictor of CVD morbidity and mortality. Indeed, both last American and European guidelines in primary prevention have endorsed using CAC as an enhancer of risk when there is uncertainty about statins' indication. However, CAC testing requires assessment by a radiologist and it has a cost that is not still reimbursed by several medical insurances in Latin America. Moreover, in young female individuals (<50 years), the probability of finding a high CAC score is very low. For such a patient, a clinician could request an exercise stress test or other biochemical parameters parameter (e.g., hsCRP)^30^ to help classify CVD risk better. In this regard, maximal exercise stress testing screens for CVD, monitors BP and chronotropic response to exercise, and measures CRF.^21^ It is also a testing modality that is relatively easy to administer, non‐invasive, low cost, and safe, and it is readily available in all cardiology clinics. It is important to say that some cardiologists and patients would not agree stress testing is easy to perform in the clinical practice, as it is for example the six‐minute walking test (6MWT). This one is a useful tool for HF patients and patients entering a Cardiac or Pulmonary Rehab program to evaluate the progression or regression of symptoms and also for prognosis in those patients. Nevertheless, 6MWT corresponds to submaximal exercise and perhaps it more closely approximates the capacity to perform activities of daily living. Therefore, it is not useful in populations like ours, who did not have previous CVD or pulmonary disease, and, by contrast, had better CV performance, that is, moderate‐intense performed exercise. In this regard, the best tool to measure aerobic capacity is the cardiopulmonary stress test, with the measurement of peak VO_2._ Our population was followed in an ambulatory setting in a primary prevention program. VO_2_ testing only is performed when asked by the cardiologist in special populations, that is, HF or NYHA class III entering Cardiac Rehabilitation or pre heart transplant.^38^

To our knowledge, there has been only one study showing a relationship between altered stress test and CVD risk prediction. However, in that study, they did not add CRF to SCORE risk score to define improvement in risk prediction by CRF, but only demonstrating that both predicted CVD‐related and all‐cause mortality.^39^

Our results indicate that CRF was helpful in both males and females with a low/intermediate ASCVD risk score. In these individuals, someone who had a CRF < 9 METS had a higher risk of death in 9 years. Having this information could spare the realization of CAC scoring. Also, due to the enormous prevalence of free‐time leisure sedentarism in the population, a low CRF could help to encourage regular aerobic physical activity which is lacking in most Latin American countries.

Finally, our results demonstrated that SCORE and ACC/AHA risk scores statistically significantly determined CVD‐related mortality. This is important as these are currently the most used risk scores throughout the world. We demonstrated that the addition of CRF to these CV risk prediction models improved more their ability. Therefore, having good discrimination of CVD‐related death underlines that the “old and simple” CRF, determined by a stress test, still has a space in determining risk.

### 4.1 Limitations

Our study has limitations. Given that the subjects included in this study are those who attend a primary CV prevention unit looking for better health, there is a potential for sample bias. Second, we only report on mortality (both all‐cause and CVD‐related) since there is no reliable registry of CVD events (nonfatal myocardial and cerebral infarctions). Moreover, we did not separately report in men and women, given that in the last, only a few deaths occurred. Lastly, CRF was estimated by METS, which is an approximation of an individual's actual maximum oxygen consumption.

## 5 CONCLUSIONS

In conclusion, this study demonstrates that CRF is a good and independent predictor of CVD‐related mortality, superior to accepted CVD risk scores, and when added to standard risk scores it significantly increased their predictive power in our Chilean Latino population. It would be desirable that current American and European guidelines make it clear that CRF is still a valuable tool in the cardiologic armamentarium.

## CONFLICT OF INTERESTS

The authors declare no potential conflicts of interest.

## AUTHOR CONTRIBUTION

Mónica Acevedo and Giovanna Valentino were involved in the conceptual design, analysis, and interpretation of the data. Carlos Navarrete was involved in statistical analysis and interpretation. Ramón Corbalán was involved in the conceptual design and interpretation of the data. Lorena Orellana, Fernando Baraona, Mónica Acevedo, and María José Bustamante were involved in the acquisition and interpretation of the data. Mónica Acevedo and Giovanna Valentino drafted the manuscript. All authors revised the manuscript critically for important intellectual content, and approved the final version for publication.
